# Identity development and adaptation in adolescents with genetic conditions: a qualitatively oriented mixed-methods study to develop strategies for optimizing clinical genetics services

**DOI:** 10.1186/s13023-025-03968-x

**Published:** 2025-08-21

**Authors:** Tasha Wainstein, Cyrus Boelman, Connie Ens, William T. Gibson, Kevin Gregory-Evans, Olubayo U. Kolawole, Sheila K. Marshall, Kathryn Selby, GenCOUNSEL Study, Bartha Knoppers, Larry D. Lynd, Alivia Dey, Shelin Adam, Nick Bansback, Patricia Birch, Lorne Clarke, Nick Dragojlovic, Jan Friedman, Debby Lambert, Daryl Pullman, Alice Virani, Wyeth Wasserman, Ma’n Zawati, Jehannine Austin, Alison M. Elliott, Jehannine Austin, Alison M. Elliott

**Affiliations:** 1 Department of Medical Genetics, Faculty of Medicine, The University of British Columbia, Vancouver, BC Canada; 2 BC Children’s Hospital Research Institute, Vancouver, BC Canada; 3 Division of Neurology, Department of Pediatrics, Faculty of Medicine, The University of British Columbia, Vancouver, BC Canada; 4 Pediatric Partnership Heart Program, Pediatric BC Inherited Arrhythmia Program, BC Children’s Heart Centre, Vancouver, BC Canada; 5 Department of Ophthalmology and Visual Sciences, The University of British Columbia, Vancouver, BC Canada; 6 School of Social Work, University of British Columbia, Vancouver, BC Canada; 7 Division of Adolescent Health and Medicine, Department of Pediatrics, Faculty of Medicine, University of British Columbia, Vancouver, BC Canada; 8 Department of Psychiatry, The University of British Columbia, Vancouver, BC Canada; 9 BC Women’s Health Research Institute, Vancouver, BC Canada; 10 Department of Human Genetics, Faculty of Medicine, McGill Centre of Genomics and Policy, McGill University, Montreal, QC Canada; 11 Faculty of Pharmaceutical Sciences, University of British Columbia, Vancouver, BC Canada; 12 Centre for Health Evaluation and Outcome Sciences, Providence Health Research Institute, Vancouver, BC Canada; 13 Provincial Health Services Authority, Vancouver, BC Canada; 14 School of Population and Public Health, University of British Columbia, Vancouver, BC Canada; 15 Centre for Advancing Health Outcomes, University of British Columbia, Vancouver, BC Canada; 16 Collaboration for Outcomes Research and Evaluation, Faculty of Pharmaceutical Sciences, University of British Columbia, Vancouver, Canada; 17 National Rare Disease Office, Mater Misericordiae University Hospital, Dublin, Republic of Ireland; 18 Faculty of Medicine, Centre for Bioethics, Memorial University, St. John’s, NF Canada

**Keywords:** Adolescents, Disability identity, Genetic conditions, Genetic counselling, Psychological adaptation

## Abstract

**Background:**

Genetic counselling for adolescents necessitates an approach distinct from that used with adults. Developing best practices is crucial, considering the growing number of disabled adolescents worldwide and increasing use of genomic testing early in life. We investigated perceptions of adolescents (10–19 years) who had been diagnosed with a genetic disorder in terms of how they describe receiving, understanding, and living with a genetic condition. We undertook a cross-sectional, qualitatively oriented mixed methods study underpinned by the pragmatic paradigm. Adolescents completed two self-report measures – the psychological adaptation scale (PAS) and the illness identity questionnaire (IIQ)—and participated in semi-structured interviews. Demographic, PAS, and IIQ data were analyzed using descriptive statistics. We used phronetic iterative analysis to interrogate interview data. Qualitative and quantitative components were integrated through abduction.

**Results:**

Eighteen participants (median age: 15.5 years; 11/18 women/girls; 13/18 typical cognition; 8/18 de novo presentation) with a variety of genetic conditions participated. Participants had a mean PAS of 3.07 ± 0.84 indicating adequate adaptation. Their IIQ profiles indicated slightly better mean adaptive scores (3.10 ± 1.06) than mean maladaptive scores (2.85 ± 0.99). We developed a conceptual model that describes disability and genetic identity development and psychological adaptation among participants composed of three interacting components: internalizing processes; variability arising from contextual factors; and external factors associated with the processes. Adolescents generally moved among four internalizing processes (initiating, minimizing, exploring, and accepting). Movement across these processes took place frequently because of contextual factors like setting and disability type. Communication and engagement with caregivers, peers, and healthcare professionals, social interactions with others who have the same or a similar condition, and the impact of ableism constituted the main external factors with which adolescents engaged in the development of these identities.

**Conclusions:**

Our findings present a foundation upon which to develop a care model optimized for the needs of adolescents with genetic conditions. Enhancing access to genetic counselling as a means of facilitating identity development is an important component of these care models.

**Supplementary Information:**

The online version contains supplementary material available at 10.1186/s13023-025-03968-x.

## Background

Over the past several decades substantial increases in population sizes have resulted in an increase in the number of adolescents, including those with disabilities [1]. Recent data from UNICEF and the Global Burden of Diseases, Injuries, and Risk Factors Study (GBD) estimate that between 10.1 and 11.3% of all children have moderate-to-severe disabilities globally [2]. Over the same period, there has been an explosion in the use of genetic and genomic testing as a clinical diagnostic tool [3]. Consequently, there are likely to be increasing numbers of adolescents with confirmed genetic conditions among the population of disabled individuals.

Genetic testing has implications not only for the individual being tested, but also for family members. However, there is a preponderance of research regarding the experiences and perspectives of parents whose children have undergone testing [4–7], while far less attention has been paid to the experiences of those children [8], or their siblings [9]. Consequently, clinical genetics services (in particular, genetic counselling) may not be optimally serving the needs of adolescents with genetic conditions. In fact, when genetic testing is conducted at very young ages, it is far more common that the responsibility of disclosing the genetic diagnosis to the child will fall on parents or caregivers at some point in the future, even if they do not feel confident to do so [10, 11].

Genetic counsellors are invested in assisting people with adaptation to the implications of their genetic conditions [12] and are well positioned to attend to the psychological concerns which are concomitant with genetic testing, having a genetic condition, or being at risk to develop a genetic condition. However, genetic counsellors have reported feeling less well-equipped to provide genetic counselling to adolescents [8, 13], in addition to experiencing discomfort in engaging individuals with disabilities [14–16]. We previously undertook a scoping review of the literature to understand the ways in which adolescents have engaged with genetic counselling [8]. Our results indicated that substantial emphasis has been placed on assessing adolescent knowledge of their conditions, communication of genetic information within families, and on decision-making about genetic testing for adolescents who may be at risk of adult-onset conditions. The lived experiences of adolescents with genetic conditions constituted a considerably smaller focus of the examined literature. The scoping review highlighted that re-evaluating the genetic counselling process for adolescents would allow for its optimization.

The idea that adolescents represent a unique population who require specialized, developmentally appropriate healthcare services is well established [17]. Adolescents undergo significant physical and cognitive maturation, have specific healthcare concerns, engage in the process of identity formation, and must navigate the transition toward independence. Adolescents are also developing capacity which recognizes that as they mature, they acquire agency and responsibility for decision-making [18–20]. The cognitive flexibility of adolescents makes them particularly amenable to health care interventions designed to improve long-term health [17]. Living with a genetic condition can have profound effects on children’s health and psychological well-being, and their conditions may cause disruptions in family, social, educational, and vocational domains. Adolescents are also subject to the impacts of societal attitudes and beliefs that stigmatize disability [21]. A thorough grasp of the lived and living experiences of adolescents with genetic conditions could provide a valuable theoretical foundation for adapting genetics services to cater to the requirements of all adolescents who might find genetic counselling beneficial.

To begin building an evidence-informed genetic counselling process tailored towards adolescents with genetic conditions, we aimed to describe their experiences of identity development and psychological adaptation. We wished to understand how adolescents make sense of their experiences with their genetic conditions and how they describe receiving, understanding, and living with their genetic conditions.

## Methodology

A comprehensive description of our methodology is available (see Additional File) and aligns with “Big Q Qualitative Reporting Guidelines [22].

### Participants and recruitment

English-speaking adolescents between 10 and 19 years, living in Canada, who have a confirmed clinical or molecular diagnosis of a genetic condition were eligible to participate in this study. We recruited participants who had previously participated in the Clinical Assessment of the Utility of Sequencing as a Service (CAUSES) study [23], and research conducted by JA’s team. Recruitment sessions were also conducted for healthcare professionals at several specialty clinics within BC Children’s and Women’s Hospitals who then facilitated referrals to the study. Treating physicians who had long-term engagement with the adolescents facilitated referrals of those who they deemed capable of meaningful engagement with the interviews and completion of self-report measures used in this study. [21].

Adolescents and their caregivers were invited to discuss study participation in a “pre-interview” prior to study interviews taking place. The pre-interview facilitated psychological connection with the research team, a space for participants to ask questions about the study and express their preferences regarding logistics; determined the need for accommodations, and allowed participants to specify whether caregivers would be present for the interview [24]. When caregivers were expected to be present for interviews, we used this pre-interview meeting as an opportunity to teach them the scaffolding method (a way of helping to facilitate their child’s responses, rather than having them answer on their child’s behalf [24]). Participants were given the opportunity to select their own pseudonyms to protect their anonymity. Additionally, to ensure that participants could not be identified, we opted to report a condition category which reflected the major organ or system impacted by their genetic conditions rather than the name of the condition.

## Research team positionality and methodological framework

Our collective perspectives and identities provide an important contribution to our subjective, partial, and situated analysis. We encompass multiple generations (Gen X, millennials), gender identities (cisgender, agender), and a range of backgrounds including both Canadian-born and immigrants (South Africa, UK). The team comprises a diverse group of experienced researchers and clinicians with respect to areas of academic study and clinical practice (genetic counselling, medical genetics, social work, neurology, ophthalmology, cardiology). As such, we position this work within a “clinical epistemology” [25] which focuses on operationalizing knowledge rather than pursuing it for purely theoretical interests. This aligns with our choice of a pragmatic paradigm as the philosophical framework for this cross-sectional study [26, 27]. We used a convergent [27] and qualitatively-oriented [28] mixed methods design. Amongst the team, there are those who have a personal or family history of disability/neurodivergence. This manifested in designing a research study that centered participant voices, inclusivity, empowerment, accessibility, and flexibility.

### Qualitative study design and data analysis

Semi-structured interviews took place between participants and TW using the Zoom video-conferencing platform (https://www.zoom.us) or in-person according to participant preference. An interpretive description framework was used for the qualitative component [29–31]. As interpretive description is a pragmatic approach, it allows for exploration of research questions with a focus on practical implications within a discipline-specific context [31]..

### Data generation

We used an interview guide (see Additional File) that we developed based on a scoping review of the literature [8] and the research questions to facilitate discussion. Our approach to interviewing adolescents with genetic conditions was carefully considered to mitigate power imbalances and optimize their feelings of comfort and control [24, 32]. Interviews were audio-recorded and transcribed verbatim (by either TW, a professional transcription service, or a research assistant) and were checked for accuracy against the original audio recordings. Transcripts were imported into NVIVO (Release 1.7.1; https://lumivero.com) which was used for basic organization and recording of primary-cycle codes and their descriptions.

### Data analysis procedure

We used a phronetic iterative approach to analyze the data [33, 34]. After immersion in and familiarization with the data through repeated readings of the interviews and reviewing field notes taken after each encounter, TW completed inductive primary-cycle coding for all transcripts. [35] Initial interpretations of the data were discussed and refined through secondary-cycle coding and collaborative analysis [35] among TW, JA, and AME. Synthesizing [33] was achieved through regular debriefing meetings after each interview and between each stage of the phronetic iterative analysis, the use of field notes and analytic memos, and critical reflections of the ways in which our positionalities and values were influencing our interpretations. The iterative and recursive analysis process and regular meetings allowed for the development and refinement of a conceptual model. We used information power to evaluate the adequacy of our participant group [36].

### Quantitative study design and data analysis

In addition to their interviews, participants completed a brief demographic questionnaire (see Additional File) that included information about age, gender, ethnicity, and genetic condition, as well as two self-report questionnaires. These questionnaires were selected to provide additional insight into adolescents’ overall lived experiences because of their relevance to identity formation and adaptation. All quantitative data were collected and managed using REDCap electronic data capture tools [37, 38] hosted at BC Children’s Hospital Research Institute. Demographic information as well as the outcomes of both instruments are reported with respect to their measures of central tendency and distribution.

### Psychological adaptation scale

The first instrument was the Psychological Adaptation Scale (PAS) which assesses emotional and cognitive aspects of coping and adaptation to a condition at a given time [39]. Mean (± standard deviation) scores were calculated for each sub-scale (ranging from 1 to 5). An overall score was also calculated by averaging responses across all four dimensions; a score of 3 is considered to reflect adequate adaptation [40], and the higher the score, the higher the level of adaptation to the condition. Cronbach’s alpha (α) was calculated for the total mean PAS as well as each sub-scale. Prior use of the PAS has predominantly evaluated the construct in adults or adult caregivers of children with genetic conditions with results indicating high validity and reliability in populations with neurofibromatosis [39], Rett syndrome [41], bipolar disorder [42], Down syndrome [43], and neuromuscular diseases [40]. PAS has also been measured in adolescents with Klinefelter syndrome [39, 44].

### Illness identity questionnaire

The second instrument was the Illness Identity Questionnaire (IIQ) which measures the degree to which a condition influences identity formation [45–48]. Mean (± standard deviation) scores were calculated for each sub-scale (ranging from 1 to 5), with higher scores reflecting greater congruence with the dimension. When combined, mean scores of the rejection and engulfment sub-scales provide insight about maladaptive illness integration, while the mean scores of the acceptance and enrichment sub-scales provide insight about adaptive illness integration [49, 50]. Cronbach’s alpha values were calculated for the four IIQ sub-scales to evaluate internal reliability and consistency in this participant group. The IIQ has shown high levels of validity and internal reliability in populations of adolescents, youth, or emerging adults with type 1 diabetes [46], congenital heart disease [49], neuromuscular disorders [51], celiac disease [52], and refractory epilepsy [50].

### Integration of qualitative and quantitative data

An appropriate way of integrating the qualitative and quantitative analyses emerged by abduction [53, 54], a creative inferential process through which we perceive relationships between phenomena and observations. We undertook exploratory inferential statistical analyses to interrogate relationships between our conceptual model and scores from participants’ PAS and IIQ surveys. As our data did not meet assumptions of normality or homogeneity of variance required for parametric tests (i.e., ANOVA), we used the Kruskal–Wallis test, a non-parametric method, to compare medians across the four internalizing processes outlined in the conceptual model. In instances where significant differences were identified, Wilcoxon rank- sum tests were used to determine which process differed from each other. Statistical significance was defined at alpha level < 0.05.

## Results

### Participant demographics

Eighteen participants completed the PAS and IIQ surveys and participated in interviews. Table 1 lists participant demographic and genetic testing information. Participants ranged in age from 10 to 19 years (median: 15.5 years; IQR: 13.75–17 years). At the time of the study, all participants had a clinically confirmed diagnosis of a genetic condition and 15/18 had a confirmed molecular genetic diagnosis. Two participants were siblings with all remaining adolescents being unrelated to any other study participants. There was extensive heterogeneity in terms of the genetic conditions that were represented: all participants had distinct conditions apart from two unrelated adolescents with the same microdeletion syndrome and the two siblings with the same cardiac condition. With respect to cognition, 13/18 were typical (i.e., their ability to think and reason was aligned with what is typical for their age). Of five adolescents who opted to have a caregiver/parent present for their interview; 3 had atypical cognition and 2 had typical cognition.

**Table 1:** Participant demographics and genetic testing information (*n* = 18)

Variable	N
*Age (years)*	
10–-14	5
15–19	13
*Self-reported Gender*	
Woman/girl	11
Man/boy	7
*Self-reported Ethnicity/ies*^*1*^	
White; “Caucasian”; Canadian; European	15
Trinidadian; Korean; “Arabic” (1 each)	3
South Asian / “Brown”	2
Not reported	2
*Condition Category*	
Cardiac condition	3
Neuromuscular condition	2
Microdeletion syndrome	2
Ophthalmologic condition	2
Muscular dystrophy	2
Neurological condition	1
Overgrowth syndrome	1
Hematological condition	1
Congenital malformation syndrome	1
Nervous system condition	1
Neurodevelopmental condition	1
RASopathy	1
*Genetic Test Type*	
Single Gene	6
WES	6
Targeted Gene Panel	4
FISH	2
*Genetic Test Outcome at Time of Interview*	
Positive	15
Negative	1
Pending	1
Probable	1
*Affected Family Members*	
Yes (inherited)	10
No (de novo)	8
*Cognitive Development*^*2*^	
Typical	13
Atypical	5

^1^Participants could report as many ethnicities as relevant, reflected in a total response rate of greater than 18. ^2^As determined by the referring clinician/caregiver. FISH: Fluorescence in situ hybridization; WES: whole exome sequencing

### PAS and IIQ outcomes

Overall, study participants had a mean total PAS of 3.07 ± 0.84 (range: 1.55–4.42) indicative of adequate adaptation at the time of survey completion. A combined mean score for the maladaptive component of the IIQ (rejection and engulfment) among the group was 2.85 ± 0.99 and the adaptive component (acceptance and enrichment) was slightly higher at 3.10 ± 1.06. PAS and IIQ sub-scale outcomes and internal reliability scores are summarized in Table 2. Densities and distributions of both the PAS and IIQ outcomes can be found in Fig. A1 (Additional File). We did not discern any consistent patterns with respect to the interacting components of the conceptual model and participant ages or types of conditions.

**Table 2:** Psychological adaptation scale (PAS) and illness identity questionnaire (IIQ) mean sub-scale and internal validity outcomes

Sub-scale	Mean (± SD)	Cronbach’s alpha (α)
*PAS*		
Coping efficacy	3.20 (± 0.92)	0.689
Self-esteem	3.18 (± 0.89)	0.839
Social integration	3.20 (± 1.00)	0.834
Spiritual well-being	2.71 (± 0.97)	0.822
*IIQ*		
Rejection	2.77 (± 0.71)	0.627
Engulfment	2.05 (± 0.68)	0.824
Acceptance	3.72 (± 0.76)	0.783
Enrichment	3.52 (± 0.84)	0.921

**Fig. 1:**
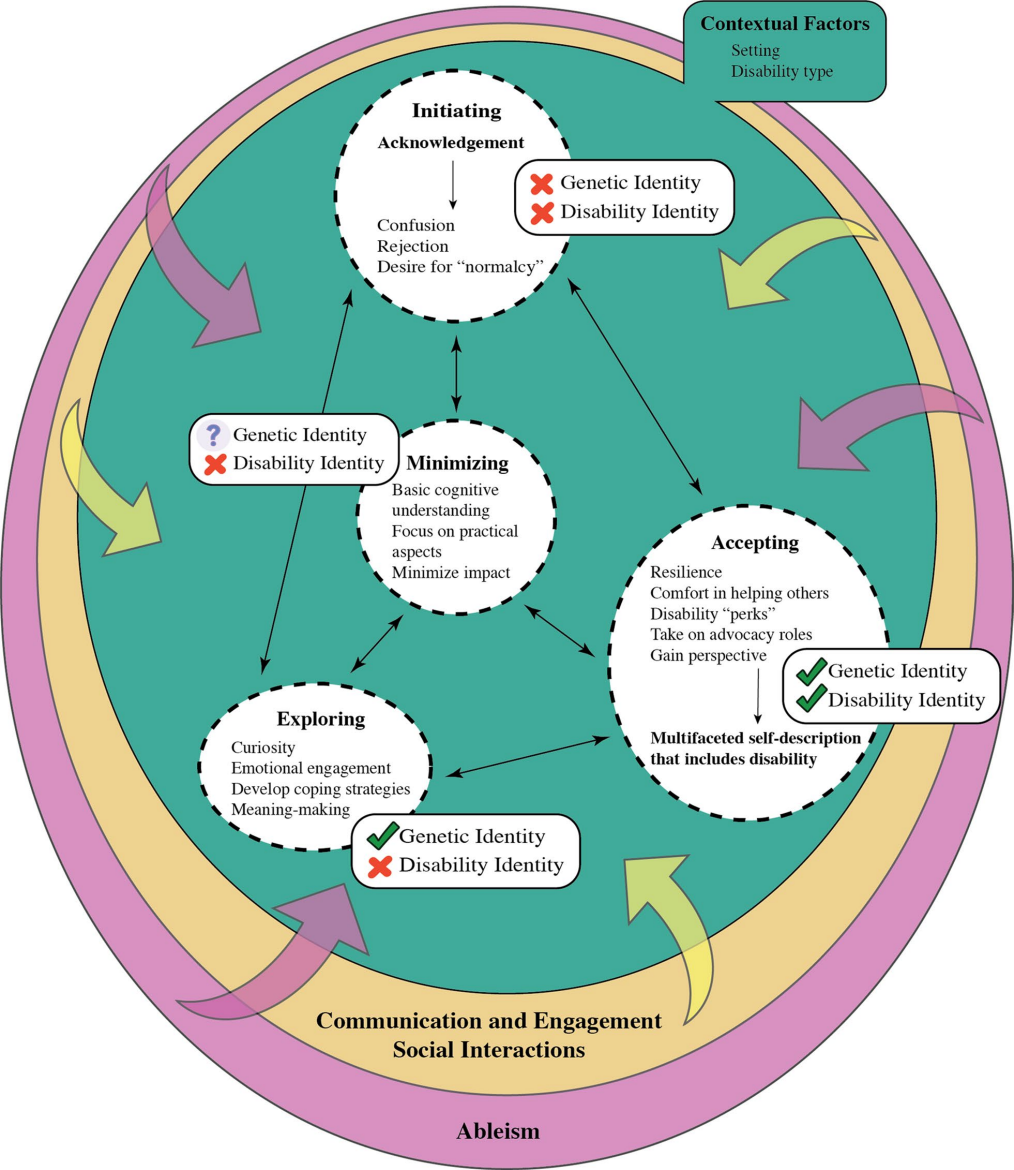
Conceptual model of genetic and disability identity development and psychological adaptation in adolescents with genetic conditions

### Conceptual model of genetic and disability identity development and psychological adaptation

Our conceptual model (Fig. 1; Table 3) describes identity development and psychological adaptation which the adolescents with genetic conditions in our study experience. With respect to identity development, the model incorporates both identity as a person with a genetic condition and disability identity. For the sake of brevity, this is referred to as “genetic and disability identity development”. We have organized our conceptual model into three key components, (i) internalizing processes of genetic and disability identity development and psychological adaptation, (ii) variability that arises because of contextual factors, and (iii) external factors associated with these processes. We understand ‘genetic identity’ as the integrative process of incorporating genetic information into one’s ongoing life, and to construct a coherent, meaningful sense of oneself [55]. Disability identity can be described as “a sense of self that includes one’s disability and feelings of connection to, or solidarity with the disability community” [56].

**Table 3:** Representative quotations reflecting the conceptual model of genetic and disability identity development and psychological adaptation in adolescents with genetic conditions

Conceptual model component	Representative quotation(s)
*Internalizing Processes: Initiating*	
Acknowledgement	“In elementary school, I had really sharp, pointy teeth. And so, I thought that was cool, I thought it was like a vampire. But then, when everyone started to lose teeth and grow adult teeth, only some of my baby teeth were loose and only some of my adult teeth came in. And so that’s the main time when I realized that it was different.” [Joey, 15, he/him, overgrowth syndrome]
Confusion	“My little brain had no idea what was going on with me and I just was very confused.” [James, 16, congenital malformation syndrome]
	“I think it’s weird that my dad would have the gene, but it’s not affecting him. So, it was a bit confusing, I guess, for him to be like, normal.” [Rory, 15, cardiac condition]
Normalization	“I think I’m kind of glad that I was diagnosed so young. So, I got to grow up learning how to do it [adapt] instead of learning how to do it when I was grown up. I think it’s a lot harder in that sense.” [Chloe, 17, she/her, ophthalmological condition]
*Internalizing Processes: Minimizing*	
Basic cognitive understanding	“I know what it is and that’s kind of all I need to know.” [Ali, 13, she/her, cardiac condition]
Focus on practical aspects	“[mother addressing her son]: So, do you think about it on a daily basis or only when we have an appointment? [he nods] Only when we have an appointment. So basically yeah, he doesn’t dwell on it.” [Spider-man, 11, he/him, microdeletion syndrome]
Minimize impact	“I kind of feel guilty for saying I have [name of condition]. A lot of people have it a lot worse than I do. And so, it just feels kind of weird to explain that I have it. Because I feel like I am taking away from people who have it more serious than I do.” [Lily, 19, she/her, RASopathy]
	“It exists, but it’s not a huge thing. It’s kind of just a nonissue sort of thing. It’s there, I’m here. We just kind of coexist.” [Sourdough, 16, he/him, cardiac condition]
*Internalizing Processes: Exploring*	
Curiosity	“It helped me validate what I was feeling. I am pretty sure I have ADHD. And I was like, what if that’s related? So, I went and did some research, and I found a research article that explains a majority of kids with [name of condition] also have been diagnosed with ADHD. So, I was like, OK, that makes a lot more sense of why I am feeling this way.” [Lily, 19, she/her, RASopathy]
Emotional engagement (fear)	“Not everything goes to plan, and like my health could get worse… or better. Which, I’m hoping it does get better. But it could always go south, and just drastically change over like the next 5, 10, years and just go really bad. Which I’m not hoping for, so…” [James, 16, he/him, congenital malformation syndrome]
Developing coping strategies (reframing)	“I guess the fact that I can still play sports [helps her cope with the reality of her condition]. Like, it could have been way worse; it could have had a way bigger impact on my life.” [Ali, 13, she/her, cardiac condition]
Develop coping strategies (maladaptive)	“I also feel like whenever I have a big trauma, my brain kind of blocks it out and I don’t remember what happened. So, I feel like all those traumas have been protecting me from remembering, because I’ve had a lot of traumas. I don’t really quite remember… I mean I’ve heard the stories though and seen the pictures.” [Adelaide, 16, she/her, microdeletion syndrome]
Meaning-making	“I feel like it’s kind of brought us a bit closer together, because we have this shared thing that we all have to deal with, and we can all kind of vent to [each other] about.” [Ali, 13, she/her, cardiac condition]
	“I want to be a therapist. I mean that involves a lot of talking – and listening – so that’s good for me. And I think having a disability might make it easier for some people because they see that I know hardship. So, they might feel more comfortable.” [Chloe, 17, she/her, ophthalmological condition]
*Internalizing Processes: Accepting*	
Resilience	“I think I got better as the last couple [hospitalizations] came on, because it didn’t take me as long to get back into my skills, I’d say the third one from the first one. And now whenever we have a bunch of time off, I feel like I only need a week to get all my stuff back because I’m so used to it. With the pandemic, we had a couple of months off practice and had to do Zoom practice. When we went back, I actually didn’t find it too difficult to get all my skills back, because it was something that I’ve already done. I think it made it a little bit, I wouldn’t say easier, but I did have a pretty easy time getting everything back with COVID, like it wasn’t a big deal. [Adelaide, 16, she/her, microdeletion syndrome]
	“I feel like it made me grow as a person more. It makes me feel like I already accomplished something really cool. Like, it’s not really accomplishing it, but, showing me that I’m strong and I can get through things.” [Layla, 16, she/her, hematological condition]
Comfort in helping others	“Because of my condition, sometimes I go through things that not everyone else goes through. So, I use it as a blessing for me. I think it gives me the tools to help people.” [Aaron, 14, he/him, muscular dystrophy]
	“People are all different, and they go through things at home, or just in their daily life, or struggle at school. School can be really hard on someone – just like me…I get a lot of satisfaction from helping people, because that’s who I am.” [James, 16, he/him, congenital malformation syndrome]
Disability “perks”	“If you really didn’t want to do something, you’d just say, ‘Oh, I can’t do it, I’m blind’. Yeah, sometimes you can use it to your advantage.” [Chloe, 17, she/her, ophthalmological condition]
Take on advocacy roles	“Maybe like educating people on it? Maybe do a blog post about it and then get a bunch of other people with genetic conditions. It doesn’t just have to be [specific condition]. And get everybody else’s experiences so that younger kids can see what it’s like, what their future could look like.” [Lily, 19, she/her, RASopathy]
*Contextual Factors: Setting*	
Medical challenges highlighted	“Sometimes, I like to think I’m a normal kid. But whenever I have a small thing that should be small for other people, it’s not so small for me. Like if I get a big cut on my hand or something, for other people, [they would just be] bandaged up, but for me, I have to take antibiotics so that I don’t get an infection. No normal kid would hide behind the couch because they don’t want to go to hospital, or they wouldn’t avoid trying to go.” [Adelaide, 13, she/her, microdeletion syndrome]
Participation in sports	“It’s like a pathway to another dimension. It’s just like all the fears, and pain, and agony just disappear and it’s really nice.” [James, 16, congenital malformation syndrome]
	“Because I can’t walk, and I can get tired or get pain or get sick more than most people do. But then the hardest part is I can’t participate in all the activities that people my age do, like sports.” [Aaron, 14, he/him, muscular dystrophy]
Labelling	“I just feel like it might be different for other people with the same condition… because of the circumstances of everyone’s life. We all have different things that we do in our lives, so if you were to ask someone else about their lives, everything is different [even though they have the same diagnosis].” [Nasreen, 13, she/her, ophthalmological condition]
*Contextual Factors: Disability Type*	
Apparent disabilities (sense of belonging)	“[mother addressing her daughter]: So, it was nice to spend time with them? Did it feel more comfortable to be around a lot of people who are also using wheelchairs compared to school where you’re maybe only one of two or three people using a wheelchair? [daughter responds]: Yeah.” [Opal, 10, she/her, neuromuscular condition]
Non-apparent disabilities (sense of exclusion)	“Like say for example gym class, they would ask me to stand on that [balance beam], where the gym teacher forces me in front of the whole class. Whatever- I know I’m not going to be able to do that. So, I just don’t stand on it. So, I’m trying to avoid stuff I can’t do. I mean it’s not a problem unless I kind of make it a problem or do an activity that I know it would be a problem..” [Jessie, 18, she/her, neurodevelopmental condition]
Non-apparent disabilities (forced disclosure)	“I can pass off as not having a genetic condition. So, people think I’m normal in a sense.” [Lily, 19, she/her, RASopathy]
*External Factors: Communication, Engagement, and Social Interactions*	
Reliance on mothers	“I like to have her [mother] explain things because sometimes I feel like she explains it better than I would do, because I tell her everything, so she knows it. I think there are instances where I tell them, but I do prefer to have her say it.” [Adelaide, 16, she/her, microdeletion syndrome]
Differential treatment	“I’d tell all my teachers I couldn’t go on these field trips because my mom was like, ‘no, you’re not allowed’. Like she really wanted to put me in a bubble… because if I got even a little bit hit, it was really scary. She protected me a lot more than [sibling], she was very careful about me, and I was always kind of sad that [sibling] got to do more opportunities in [sports] than I did.” [Layla, 16, she/her, hematological condition]
Communication with healthcare professionals	“He [doctor] explained it more and more as I got older. Because there is just some stuff that I was just too young to talk about. At my last appointment, he explained that if I were ever to get pregnant, how that would work, because that was when I was 18. So that’s when I was able to fully understand. So, I think that helped me a lot. In the moment when I was diagnosed, just explaining what would be further steps in that moment. And then, once I got to a certain age, explaining more steps.” [Lily, 19, she/her, RASopathy]
	“It didn’t really explain that much. They might have given it [further information] to my mother” [Jessie, 18, she/her, neurodevelopmental condition]
Engaging with peers with the same or a similar condition	“It’s nice to chat with someone who knows what you’ve been through versus someone who doesn’t know, if you’re actually going through a hard time.” [Adelaide, 16, she/her, microdeletion syndrome]
Engaging with peers with other types of medical complexity	“It’s quite nice to have a friend that you can talk to about this kind of stuff”. [Maya, 15, she/her, neurological condition]
Rarity of condition obstacle to peer support	“If I had the chance, I would obviously do it. But it’s pretty rare to find someone like me.” [James, 16, he/him, congenital malformation syndrome]
Reluctance towards peer support	“I think because it’s such a minor thing, I don’t really think that matters to me. Especially because it affects people so differently. If I was to talk to someone who was actually really affected by it, I feel like I wouldn’t benefit from that.” [Rory, 15, she/her, cardiac condition]
*External Factors: Ableism*	
Bullying	“I’ve got really made fun of because they’re [peers] like, ‘well, why are you only swimming four times a week when everyone else is swimming seven’? They’d tell me to ‘just suck it up’ or make fun of me for it.” [Layla, 16, she/her, hematological condition]
Dismissiveness	“My coach kind of thinks it’s not really real, and it’s kind of hard to explain it to him. When I try to explain it, that’s just kind of annoying, because it’s really hard to explain it without trying to make it like an excuse. Like when I was little, it always sounded like an excuse. I don’t look sick or anything. So, if someone wants to know, I have to give them the whole background, the whole 20-min story…. So, I really have to explain it to them and go into detail if I want someone to understand what I’ve been through, which is kind of annoying because you don’t just see me and think, ‘oh, yeah’.” [Layla, 16, she/her, hematological condition]
Limitations of societal knowledge	“When people ask, I’m like, ‘oh, it’s just something to do with the misspelling of my gene, kind of like Down syndrome.’ Which they get, they know what that is.” [Lily, 19, she/her, RASopathy]
Infantilization	“They don’t say anything mean, but it’s the way they talk to me. They talk to me like I am a baby. And like I need so much help, and I can’t live a normal life.” [Chloe, 17, she/her, ophthalmological condition]

### Internalizing processes

We define internalizing processes as those with which adolescents consciously or subconsciously engaged in making sense of or developing their identities. Genetic and disability identity development and psychological adaptation in adolescents with genetic conditions generally followed pathways among four processes: initiating, minimizing, exploring, and accepting (Fig. 1). Movement among these processes was reported as frequent. Therefore, although the description below details each process in apparent isolation, it is important to recognize that each adolescent may have demonstrated features of multiple processes over the course of their interviews, in addition to having broad alignment with one of the processes overall. Also, there is no hierarchy among the four internalizing processes.

#### Initiating

For the adolescents in our study, internalizing frequently began with an acknowledgement of some kind of difference or challenge (physical, health, or educational), which led to their being informed, usually by their parents, of a diagnosis/condition that had already been established. Others described first noticing differences and only then embarking on a path towards diagnosis. The experience of recognizing their difference/s was regularly associated with a feeling of confusion stemming from a lack of understanding. In a handful of noteworthy cases, confusion stemmed from a misalignment between their understanding of genetics/inheritance and their expectations of how the condition manifested in their families. For example, one adolescent described being confused why she was the only individual in her family who was found to have her condition even though other relatives had similar features.

Some participants felt unable to isolate a specific point at which they acknowledged their differences because they had been diagnosed at such a young age that the aspects which signified their differences felt “normal” to them. Normalizing language (“*It’s always been like this*”, “*I’ve grown up with it*”, “*It’s always been a part of my life*”, “*I’ve had to do it for such a long time, it’s just normal now*”) was frequently employed to indicate a relatively mild emotional response to their diagnosis. However, even if their disabilities manifested earlier and this sense of normalization was profound, this did not imply that adaptation existed automatically; grappling with the process of genetic and disability identity development still needed to take place.

Adolescents who tended to deny, reject completely, or feel uncertain towards disability identity and/or identity as a person with a genetic condition, exemplified those engaged in the initiating process (Fig. 1). This was described by one participant in response to being asked if she identified as a disabled person or a person with a genetic condition, “*No. I think I’m just someone who has one. It’s not really part of my identity. It doesn’t affect any aspect of me.”* [Rory, 15, she/her, cardiac condition].

#### Minimizing

Adolescents engaging in the minimizing process tended to have a basic cognitive understanding of their genetic conditions and did not demonstrate any desire or interest in obtaining further information. While some participants acknowledged that this may change in the future if their health care needs changed, others felt that this attitude would prevail regardless.

When considering the impact their diagnosis had on their lives, their focus was oriented towards the practical aspects of their conditions, and they frequently downplayed any emotional facets of their experiences. Several adolescents described either forgetting they had a condition on some days, or that they actively avoided thinking about it. Several participants engaging in this process emphasized that there were others with the same or a similar condition who experienced far more severe impacts. Even without comparison to others, there was an inclination among these participants to minimize the impact of their conditions.

Discussions around identity as a person with a genetic condition again resulted in a tendency to qualify this through comparison to those whom they perceived to have more serious conditions than their own (Fig. 1). For example, one participant shared, “*I guess so, yeah. There are definitely some more serious genetic conditions that people have, but I don’t really want to be lumped into that category because I don’t think I have a serious condition. So, I would definitely involve the word, ‘mild’ in there.*” [Ali, 13, she/her, cardiac condition]. The discussion of disability identity did not arise among any participants who could be categorized as engaging in the minimizing process.

#### Exploring

Adolescents who were engaging in the exploring process demonstrated curiosity towards learning more about their conditions and in several cases had sought out information beyond what had been offered to them by healthcare professionals or their parents. Others spoke of seeking out information (usually online) about the medical aspects of their diagnosis as well as a desire to understand the experiences of those who shared their conditions.

The exploring process was also exemplified by a greater willingness to engage with the emotions adolescents felt with respect to their genetic conditions. In many cases, this centered around their frustrations of finding the right medications, dealing with side effects, and the trauma associated with managing their conditions (needles used for blood draws or injections were a recurrent example). Several participants also shared their fears about the future, frequently in relation to their health. For other adolescents concern centered around their educational and vocational aspirations. Many adolescents engaging in the exploring process described the impact of their conditions as being a constant presence at the back of their minds.

When asked how they managed these emotions and experiences, adolescents provided examples of several coping strategies that they had developed. Prominent among these strategies was the use of distractions; siblings, friends, and sports were regularly cited as being useful distractions from these difficult emotions. Others sought emotional support more directly by addressing their feelings or concerns with their parents and occasionally, their friends. Having a sense of humor and being able to laugh about their experiences as well as reframing their situations were also mentioned. A few participants described a drastic shut down of memories about traumatic experiences in order to protect themselves.

Many adolescents engaging in this process began to attribute positive meaning to their experiences and recognized that they could hold value. For some, this value lay in the closeness their conditions generated within their families. For many, appreciation of their conditions was reflected in a desire to pursue a career in healthcare where they felt they could be of service to those who were like them.

Participants engaging in the exploring process tended to describe their identity as a person with a genetic condition as a core part of who they were, as one participant explained, “*It’s a part of who I am. It makes me different from other people. But it’s not something huge that dictates my life. It just makes me do things sort of differently sometimes.*” [Nasreen, 13, she/her, ophthalmological condition]. However, none of the participants who aligned with the exploring process were accepting of a disability identity (Fig. 1). Several participants qualified their responses with comparisons of their physical abilities to those whom they deemed disabled. One participant reflected, “*Not really. I know I have hearing aids, but I am able to do things, so I wouldn’t necessarily call myself disabled. It depends what you mean by disabled because there’s a couple of different meanings. But I’d call myself an active person. So, I don’t think so, no*.” [Adelaide, 16, she/her, microdeletion syndrome].

#### Accepting

Adolescents engaging in the accepting process shared examples of positive disability experiences, an indicator of adaptive genetic and disability identity development. Many adolescents described how their genetic condition had provided them with a unique ability to manage setbacks and to be resilient. Several adolescents also valued and appreciated how their own experiences could be drawn upon to be a source of comfort to others. Other positive experiences included receiving perks that would give them access to free transportation, free movie tickets, or tax benefits, as well as a plausible reason to skip school or avoid undesirable tasks. Several participants appreciated how their experiences had allowed them to gain perspectives and outlooks on life that others may not share. For example, one participant described how she was able to pay less attention to perceived trivial “*high school drama*” because of the maturity and perspective she had gained as a result of her disability. Adolescents engaging in the accepting process demonstrated a desire to be involved in advocacy, awareness, or education efforts.

Participants engaging in the accepting process tended to affirm both their disability identity and their identity as a person with a genetic condition (Fig. 1). For one participant, disability identity was simply described as, “*Yeah, it’s just who I am.*” [Ben, 17, he/him, muscular dystrophy]. Another participant reflected, “*Yes, I have a genetic condition; it’s just what it is. It’s not what I am, it doesn’t define me, but it shapes me*.” [Chloe, 17, she/her, ophthalmological condition]. Adolescents engaging in the accepting process also described specifically seeking out friendships with other disabled people. Ultimately, participants engaging in the accepting process were able to view themselves as being multifaceted, comfortably including their disability or genetic condition, rather than having it be a singular, defining characteristic.

#### Associations with PAS and IIQ

Although all participants engaged with all the internalizing processes over the course of their interviews, we interpreted a dominant engagement with one process for each participant based on the overall impression of their interviews (i.e., the process to which they showed the greatest overall alignment). This was primarily based on TW’s careful review of their complete transcripts and collaborative discussions with the core research team. These assignments were mapped against their PAS and IIQ scores (see Additional File). Kruskal Wallis testing revealed that there were statistically significant differences among the assigned processes for the coping efficacy (H = 8.076, *p* = 0.044) and social integration (H = 8.237, *p* = 0.041) sub-scales of the PAS instrument. Analysis of the total mean PAS scores according to internalizing process approached statistical significance (H = 7.767, *p* = 0.051). For the IIQ instrument, the engulfment sub-scale was the only source of statistically significant differences among the processes (H = 9.698, *p* = 0.021). Subsequent pairwise Wilcox tests for the sub-scales that demonstrated statistically significant differences using Kruskal Wallis testing, failed to identify differences between specific pairs of processes (see Additional File).

### Contextual factors

While we were able to categorize adolescents into an overall process based on the totality of their interviews, we also noted a great deal of variability and even contradiction regarding the internalizing processes. For example, a participant described initially how he felt to be the only person in his family to have a genetic condition, “*I guess kind of unique…. I’m not sure. Yeah, I guess it’s a good thing. It’s kind of hard to explain, but it’s just like, I’m the only person who has it so, I’m just kind of special like that.*” Later, when discussing whether he discloses his diagnosis to his peers, he shared, “*Well, I don’t really – because I’m not – I don’t really act different or feel different from everyone else. So, I basically just see myself as a normal kid.*” [Greg, 15, he/him, neuromuscular condition]. This idea of feeling both unique and the same as everyone else was a common juxtaposition among many adolescents. Negotiating these contradictions was an important and necessary component of the overall progression of genetic and disability identity development. We defined two important contextual factors (Fig. 1; Table 3) that appear to influence adolescents’ fluctuating processes: their setting (i.e., location or activities), and type of disability (i.e., apparent or non-apparent^1^).

#### Setting

Many adolescents described how their genetic conditions impacted every aspect of their life in some way, but particular settings or locations resulted in it taking on either diminished or greater prominence. One participant explained, “*I sort of have a daily reminder because every day there’s stuff that is hard for me to do because of my eyes. It’s not exactly something you can just forget. But it’s not at the top of my mind every day when I wake up*.” [Nasreen, 13, she/her, ophthalmological condition].

Situations in which their medical needs or challenges were highlighted tended to result in greater contemplation of their identities for many adolescents. Several participants described experiencing minor reminders when they took daily medications or needed to access health care.

Participation in extracurricular activities (especially sports) was another setting in which the significance of adolescents’ genetic conditions took on greater or lesser prominence. For some participants, these activities bolstered a sense of belonging that they did not feel elsewhere, and even felt comfortable to share their diagnoses more openly. For others, this setting served only to highlight their differences and resulted in strong feelings of exclusion.

Adolescents’ views on having a diagnosis appeared also to result in contradictory feelings according to their setting. In some locations, they were grateful to have an explanation for the challenges they had experienced or why their bodies functioned in a particular way; felt an enhanced sense of self-acceptance; and in one case felt relieved that accusations of faking symptoms for attention were proven to be unfounded. In other settings, names and labels were seen as undesirable when they served to reinforce a diagnosis as a central or defining identity.

#### Disability type

The extent to which an adolescent’s genetic condition was apparent to others was another important contextual factor that gave rise to variations in their internalizing processes. A few adolescents who had apparent disabilities, described making efforts to hide these differences (e.g., using hair to cover hearing aids, or wearing clothing that obscured physical differences). Others with apparent disabilities described experiencing a greater sense of belonging among similarly disabled peers.

For some adolescents with non-apparent disabilities, actively avoiding situations in which their disabilities might be exposed was important. For example, one participant described a greater sense of belonging in settings where her non-apparent disability could remain as such, compared to school where her need for accommodations made her feel exposed. Several participants described examples of forced disclosure in the context of having to explain repeated absences from school or extracurricular activities, or when they required assistance or accommodations.

### External factors

The final component of our model describes external factors with which adolescents with genetic conditions engage in the development of their disability identity (Fig. 1; Table 3). There were two main themes we noted in our analysis: the first is *communication and engagement* with caregivers, family, peers, teachers, and healthcare professionals, including social interactions with others who have the same or a similar condition. The second is *the impact of ableism*. Together, these themes indicate how social experiences have a substantial influence on participants’ self-construal and how they relate to their conditions.

#### Communication, engagement, and social interactions

The adolescent period is marked by increased attention to and complexity of social relationships and so it is appropriate that these factors featured prominently in the way that adolescents with genetic conditions grappled with their disability identity as well.

Primary caregivers, in particular mothers, were identified as fundamental to guiding adolescents’ understanding of their conditions. Mothers were frequently called upon as sources of information about their adolescents’ diagnoses as well as sources of support and comfort. They were the bearers of adolescents’ medical histories and on occasions when they could not provide answers to questions, would facilitate access to others who could (i.e., health care professionals, or support groups). Some adolescents explained how they would rely on their mothers to communicate needs or concerns to health care professionals on their behalf. Adolescents also reflected on their mothers’ formidable advocacy on their behalf in health care and education settings. Many adolescents did not claim to have experienced any differential treatment as a consequence of their diagnosis. However, when they did, it was frequently framed as a feeling of over-protection from their caregivers and noticeable to them in comparison to their siblings, other similar-aged peers, or for those who were diagnosed at older ages- before and after diagnosis. Some adolescents also reported noticing different treatment compared to their siblings because of aspects unrelated to their diagnosis (e.g., gender, age, birth order, and culture).

Most of the adolescents in our study reported an open style of communication within their nuclear families and consequently, their siblings were aware of their diagnoses and regularly cited as sources of support and distraction. Their relationships with their unaffected siblings were frequently perceived as being typical for any siblings regardless of the presence or absence of a genetic condition (e.g., “*we would fight a lot but that’s normal brother and sister stuff*.” [Alex, 19, she/her, nervous system condition]). One point of departure, however, were the few adolescents who described a desire to protect their unaffected siblings from the full reality of their diagnosis. In one conversation, an adolescent shared, “*well, he [brother] worries quite a lot about me.*” [Maya, 15, she/her, neurological condition]. When asked if this resulted in her choosing not to share her concerns with him, she agreed. Another adolescent described not being able to discern if her feelings of protection toward her sibling could be ascribed to her diagnosis or merely the fact that she was the older sibling. In cases where their siblings had been diagnosed with the same condition, older adolescents described taking on a guidance and education role.

Healthcare professionals were viewed as another important and trustworthy source of information about their genetic conditions, developing a sufficiently robust care relationship that adolescents felt comfortable to reach out to them directly to obtain information. Many reflected being particularly appreciative of those who offered comprehensive information about their condition in a transparent manner (including negative or painful aspects). Others stated a preference for being told information appropriate to their current needs and development, and introducing new information as it was likely to become relevant. Although less common, some participants described frustration at not being given sufficient details or not fully understanding the information that was provided. Few adolescents volunteered information about their experiences with genetics health care professionals; most recalled some details (e.g., genetic testing, the need for a blood draw, explanations of inheritance, or risk information) only when prompted. They attributed this challenge in recall to these interactions having happened a long time ago, having learned this information once they were older (from their mothers), or tacitly through other experiences within their healthcare journeys.

Few adolescents in our study described engaging with peers who had the same or a similar genetic condition. Of those who did, these interactions took place in the context of a condition-specific support group or organization and were motivated by a desire to find others who could relate to their experiences Others commented on how their participation in such groups allowed them to feel more easily understood compared to environments (like school) in which they were frequently the only person with an apparent difference; helped combat feelings of loneliness; served as a useful and reliable source of condition-related information; gave them a greater appreciation of the variability in experiences among those who had the same condition; and ultimately allowed them to interact with others like themselves. More participants spoke about their experiences interacting with peers who had disabilities or medical complexities that differed from their own (within their extended families, in other social contexts, and within the healthcare setting). Many of the benefits derived from condition-specific support groups could be found in engaging with others who had medical complexity, even though they arose from different etiologies.

We did not discern any noteworthy differences in opinions about support group involvement among participants who were the only individual in their family to have been diagnosed with a genetic condition and those who shared their diagnosis with other family members. There was marked variation with respect to adolescents’ sense of belonging as it related to participating in a support group: some described having no such feeling of belonging, others felt that it equaled their sense of belonging in groups with their nondisabled peers (especially sports teams), and yet others exclusively felt a sense of belonging in this setting.

Among those participants who had not interacted with a support group for their condition, some indicated they would be interested in doing so. They appreciated that this type of engagement would be a valuable experience, provide them with opportunities to learn more about themselves through hearing about others’ experiences, and feel understood. Despite their interest in participating, some adolescents perceived that the rarity of their conditions might be a considerable obstacle in finding an appropriate support group. The remaining participants who had not previously interacted with peers who had the same or a similar condition reported being uninterested or ambivalent about doing so. Interestingly, among this group, most explained that they felt their condition had minimal or very mild impacts on them.

#### Ableism

The entire process of identity development and adaptation in adolescents with genetic conditions occurs against the pervasive backdrop of ableism in our society. Frequently discussed by our participants were their experiences of bullying, a manifestation of ableism that is prominent in the adolescent period. For example, one participant described, “*When I was in cheerleading, we wore really short shorts. There were girls who were like, ‘oh, we’re not going to touch her ‘cause we’re gonna get whatever she has’. So, it was really hard. I was bullied and picked on a lot*.” [Alex, 17, she/her, nervous system condition]. Some adolescents described another manifestation of ableism, namely, not being believed or understood.

Many participants reflected how broad societal knowledge about genetic conditions was limited, even more so when the conditions were rare. They navigated this by providing the relevant clarifying information without waiting to be asked or comparing their condition to a more readily accessible example (e.g., Down syndrome). Poor societal knowledge was also frequently attributed to a lack of formal education about genetic conditions, such that knowledge was only gained through personal experience. Some saw this as an opportunity to increase awareness and engage in advocacy, while one participant directly ascribed this to ableism, saying, “*I think it’s this stigma around being disabled. There’s almost no representation anywhere. So, people aren’t really exposed to it. So, they don’t really understand. They just think that people with disabilities are sad little people who need help.*” [Chloe, 17, she/her, Ophthalmological condition]. Another participant acknowledged how the uncommon experience of seeing someone on a reality TV show who had a similar condition to hers would be of benefit to others, “*Because, out of all the shows, movies, stuff that I have watched, I have never seen anybody that went through what I did. I think it would help a lot of younger kids too if they also saw it.*” [Lily, 19, she/her, RASopathy].

Some participants also described being infantilized because of their condition; this seemed to be prominent among those who had apparent disabilities. For example, “*Sometimes I feel people treat me like I am younger than 14. Probably because they don’t deal with many people who are like me, so they’re unsure how to act. I think they think that’s what I want.*” [Aaron, 14, he/him, muscular dystrophy].

## Discussion

Previous research has not investigated the lived and living experiences of adolescents with genetic conditions in terms of identity development and psychological adaptation. We aimed to address this gap as a way of building a foundation upon which to develop/adapt a genetic counselling process that is optimized to their needs. Our investigations have allowed for the development of a conceptual model that expands the existing understanding of disability identity development, which has focused predominantly on adult populations [57]; only a single model has been developed for adolescents [58]. Our conceptual model describes the process of disability and genetic identity development and psychological adaptation in our participants as being composed of three interacting components: internalizing processes, variability arising from contextual factors, and external factors associated with the processes.

### Psychological adaptation and disability considerations

With respect to the experiences of adolescents whose disabilities arose from a genetic etiology and had an early onset of disability, our findings corroborate differences in this group that have been previously described compared to those who have acquired disability later in life [57–60]. Improved adaptation among those who have an early onset of their disabilities is thought to arise from an inability to compare it to a different state of functioning prior to onset [59] as portrayed by many participants in our study who used normalizing language to describe their experiences and were unable to target the specific point in time that they recognized their differences.

Results from the PAS and IIQ instruments support these findings. Although limited in our capacity to compare the PAS findings to existing literature (there are few studies which use the PAS for non-adult populations), we do note slightly higher mean scores in our population [42, 44, 61, 62]. Higher adaptive scores over maladaptive scores using the IIQ instrument have also been noted previously [46, 49, 51, 52]. Although, our investigation into differences among internalizing processes with respect to mean total PAS scores fell just short of statistical significance, it may be considered to have practical significance. This is especially noteworthy when we consider that two of the PAS sub-scales and one of the IIQ sub-scales demonstrated statistically significant differences among processes, despite the exploratory nature of these analyses, the small sample size (for quantitative analyses), and the associated lack of statistical power.

### Genetic identity

These interviews make novel contributions with respect to the concept of identity as it relates to having a genetic condition. Identification as a person with a genetic condition varied across the four processes: those engaging in the initiating process rejected a genetic identity, those in the minimizing process conditionally accepted it, those in the exploring process accepted it, and those in the accepting process endorsed it as a core part of their identity. Our conceptual model highlights how adolescents’ genetic identities are fluid and responsive to changes in their social settings. Identification as a person with a disability resulted in an additional layer of variation: for some these two identities were inextricable; for others, they constituted interconnected identities (i.e., they were distinct but related); and for yet others, they comprised separate entities that were accepted or rejected based on their own merits. Factors such as perceptions of severity and ability, support needs, societal perceptions, and individual experiences were considered when contemplating the acceptability of these identities. While previous research [55] has indicated a tendency among adults not to align genetic and disability identities with one another, our study suggests that the relationship between the two is nuanced and complex for adolescents. This is worthy of additional exploration especially with respect to the ways in which these factors may potentially affect health behaviors.

### Fluidity of genetic and disability identity development

We have emphasized the fluidity of the proposed model to demonstrate that genetic and disability identity development is not linear. Adolescents may align with more than one internalizing process at a time or may not align with one or more processes at all. In so doing, we hope to create awareness of the fact that these aspects of identity development and psychological adaptation may need to be revisited repeatedly. Further corroborating prior research [58, 63] are our findings related to the essential impact of contextual factors (specifically, setting and type of disability) on these fluctuating processes. Adolescents’ acceptance or rejection of their genetic and disability identities changed according to whether they served a sense of belonging or a sense of isolation. Learning to manage the contradictions and juxtapositions of these experiences constitutes an important component of constructing narratives about their identities [64].

Sports (gym class, extracurricular activities) was a setting in which differences manifested. A recent systematic review reported the perceived benefits of participation in sports for disabled children and adolescents [65]. These included health benefits, improved confidence, opportunities for socialization, development of a sense of belonging, a sense of normality, and the opportunity to be seen as an athlete. Healthcare professionals have been encouraged to promote participation in sports in service to these benefits [65, 66]. However, we have shown that this setting can be a source of frustration and forced disclosure of adolescents’ disability status. This suggests the need for healthcare and other professionals to recognize the multifaceted and complex process of genetic and disability identity development in these adolescents as fully as possible to provide quality services to this population. Given that settings such as school and sporting activities were areas of both support and concern for adolescents in our study, healthcare professionals can take a more balanced and individualized approach by tailoring their recommendations to meet individuals’ needs. One example might be the provision of support or documentation for adolescents like those in our study who had difficulty being believed because of a non-apparent disability.

The apparent or non-apparent nature of an adolescent’s genetic condition in our study induced variation in engagement with internalizing processes within the proposed model, similar to what has been described [58, 59]. For adolescents with apparent disabilities, the route to acceptance of a disability identity is more straightforward, encouraged by the recognition of the benefits and protective effects of engaging with the disability community. Conversely, for those with non-apparent disabilities, conforming to mainstream culture to avoid discrimination and stigmatization, appears to be the more typical societal response. Facilitating adolescents’ understanding of the non-hierarchical nature of their genetic and disability identity development may be helpful in normalizing these responses.

### External factors impacting genetic and disability identity development

Mothers represented the key source of information related to adolescents’ genetic conditions in this study, followed by healthcare professionals (genetic counsellors were rarely mentioned), in alignment with prior literature [8]. Although we did not formally assess knowledge or genetic literacy in this investigation, several instances of inaccurate genetic information were noted during interviews (e.g., overestimating risk to future offspring; incorrect inheritance), and genetics was recognized as a source of both confusion and curiosity by many participants. Clear, accurate, and developmentally appropriate information provision has been assessed as being integral to a young person’s ability to cope with and adapt to a genetic condition. Yet, evidence has shown that parents struggle with conveying this information to their children, and healthcare professionals may be less effective than ideal [10, 11, 67]. A truly optimized genetic counselling process for adolescents with genetic conditions would necessitate responding to both educational and identity aspects and should include supporting caregivers.

Our results demonstrate that genetic and disability identity development occurs within the context of sibling relationships. Adolescents described typical, close, and supportive relationships with their siblings but were also aware that their conditions impacted the nature of these relationships [9]. These influences on adolescents with genetic conditions and their typically developing siblings should be taken into consideration when designing inclusive and effective support systems.

### Impact of the oppressive system of ableism

Like other literature has demonstrated [68–70], the adolescents in this study described overt examples and experiences of ableism (i.e., bullying, dismissiveness, lack of representation, infantilization) highlighting how this oppression is deeply ingrained in our society. Society’s emphasis on health, wellness, productivity, and intelligence might compound concerns expressed by adolescents about the way in which their genetic conditions may impact their futures. Adolescents in our study alluded to a fear of being perceived as disabled as it related to an over-reliance on stereotypes about disability. In a related study, we investigated the implicit and explicit attitudes of typically developing adolescents towards their peers with genetic conditions [71]. We were interested in understanding whether improved genetic/genomic literacy could mitigate the impact of ableism on adolescents with genetic conditions. Among the 22 adolescents who participated in the study, there was a statistically significant implicit preference for non-disabled people measured using Disability Attitudes – Implicit Association Testing. There was greater diversity in their explicit attitudes discussed during focus groups and participants articulated a positive attitude towards improved genetics education. More striking, was the belief that social and personal interactions with disabled peers were essential to address negative perceptions. Together, these two studies demonstrate that improving the quality of life of adolescents with genetic conditions can only be achieved through a multifaceted approach that addresses both internal and external components of their genetic and disability identity development.

### Implications for healthcare professionals who interact with adolescents with genetic conditions

Our results have several practical implications for healthcare professionals across a range of general and specialist areas who work with adolescents with genetic conditions and their families. These implications span areas related to their medical care, emotional well-being, and development. Recognizing and understanding the complex and multifaceted nature of genetic and disability identity development in adolescents is a pivotal first step in the provision of quality healthcare services for this population. Our conceptual model is a useful tool to allow adolescents to interrogate their individual narratives as a way of making sense of their experiences and challenges in externalized, social settings. Understanding adolescents’ identity development can help healthcare professionals support them in finding and navigating their own genetic and disability identities by discussing self-perception, coping mechanisms, and personal strengths. Ensuring positive development at this stage may not only have immediate benefits for adolescents but could also serve to promote long term health and wellbeing for adults with genetic conditions.

Healthcare professionals should consider how they can work at the practical and policy levels to ensure that a safe, non-judgmental, integrated, adolescent-friendly, healthcare service in which adolescents with genetic conditions can express their concerns and have their needs addressed. This could include raising awareness, challenging stereotypes, and collaborating with patients and their families to create supportive spaces for identity exploration. They should also be effective facilitators in conversations with adolescents and their parents about disability that extend beyond explanations of the medical aspects to available support services, and strategies for managing challenges associated with their condition.

Furthermore, healthcare professionals should actively facilitate connections for adolescents with genetic conditions to support groups and services, highlighting the importance of fostering a sense of belonging within the disability community to reduce feelings of isolation and promote positive validation of genetic and disability identities [59]. In cases where a condition-specific support group is unavailable, our results indicate that the benefits of connecting adolescents with peers who have other, unrelated medical complexities remain substantial. This might be especially beneficial for those who identified the rarity of their conditions as a barrier to participating in a support group.

The adolescents in our study frequently engaged with the healthcare system and reported generally good communication and relationships with their clinicians. Given the nature of these relationships, healthcare professionals may be in a position to act as allies and advocates for adolescents with genetic conditions who may face ableism, both at the individual and systemic levels [58].

### Implications for clinical genetics healthcare professionals

Genetic counselling has traditionally served the needs of a select group of people despite our understanding of the broad impact of a diagnosis on all members of a family. Although adolescents with genetic conditions have always been theoretically able to access these services, few appear to do so [8]. The findings of this study formalize this group as an important target for genetic counselling. But there is a disconnect between adolescents who frequently rely on their caregivers for information and support and the genetic counsellors who might be well positioned and possessing the necessary psychotherapeutic skills to provide this as a service. It is imperative that genetic counsellors prioritize the development of innovative solutions to bridge this gap, including raising awareness about the availability of services for adolescents and their families. Historically, genetic services were provided almost exclusively in tertiary/academic centers, to the detriment of considerable portions of the population unable to access them. There is a growing body of evidence pointing to the successful integration of genetic counsellors into multidisciplinary teams and specialist clinics like cardiology [72], oncology [73], primary care [74], and others as a remedy for this barrier. In view of the findings of this study, we suggest that there may be an important role for genetic counsellors in adolescent medicine or other integrated youth services.

The fluctuating nature of genetic and disability identities, as described above, also suggests that the typical model of a single appointment might not meet the needs of adolescent patients whose developing maturity, autonomy, and needs will result in different questions and challenges as they grow. Genetic counselling for adolescents with genetic conditions would likely benefit from the development of an ongoing relationship across multiple sessions, as opposed to the single consultation model.

### Strengths, limitations, and future research

Our construction of a conceptual model, coupled with the use of previously validated and reliable instruments, and abduction, creates a foundation on which further work can be built to assess and measure these phenomena and design interventions. This work is further enhanced by our attention to a unique population who have not been subject to extensive prior research of this nature. Focusing on adolescents with genetic conditions extends current knowledge with respect to disability identity development, particularly regarding differences that have been noted about the timing of onset and the underlying cause of disability [58, 75].

While we evaluated the overall diversity of the adolescents with genetic conditions who participated in our study as high, we acknowledge that some aspects of identity were limited. With respect to gender, there were slightly more participants who identified as women than men, and none of the adolescents identified as non-binary. Ethnic or cultural origins reported by our participants mirrored those of the province of British Columbia [76]. We did not directly interrogate indicators of socioeconomic status (SES) such as household income, nor did we use proxy measures of SES such as Canadian postal code. Application of these findings to future research and clinical implementation should be mindful of these aspects, especially in other cultural and geographic settings. We also consider that the adolescents who assented or consented to participate (and their caregivers who consented) are less likely to represent the perspectives of those who declined to participate, especially with respect to discussing issues of identity and disability.

As there was so little existing data on this topic, we felt that including any diversity in the genetic conditions represented would meaningfully contribute to the literature. Nevertheless, some broad groups of conditions were not represented in this study (e.g., skeletal dysplasias, childhood-onset tumor predisposition syndromes). We believe that our study design and the diversity of representation we achieved, does allow for transferability to other genetic conditions and disability more generally, which has relevance for interventions and avoids siloing of knowledge [60]. Still, future studies should assess the applicability of this conceptual model in additional groups of adolescents (including those who have onset of their genetic conditions during adolescence as opposed to at birth or in early childhood) and consider evaluating whether there are any condition-specific issues with respect to disability identity development and psychological adaptation. They should also aim to recapitulate and enhance our findings through hypothesis-based statistical analyses of the PAS and IIQ measures as indicators of genetic and disability identity development. As the questionnaires were completed asynchronously, we cannot account for the degree to which caregivers may have assisted their adolescents with this task. Wherever possible, we ensured that survey links were sent directly to the adolescents’ email addresses, rather than their caregivers.

People with all types of disabilities are collectively considered to be a social minority with shared experiences in the same way as other minoritized groups [77, 78]. One difference is that individuals in other types of minoritized groups tend to share that aspect of their identity with their nuclear and extended families (e.g., racial identity). In the case of disability identity, this may not always be true [57, 59, 60, 79], as was the case for the 44% of participants in this study who were the first in their family to be diagnosed with a genetic condition. We did not perceive any notable differences in the experiences of our participants based on whether they shared their genetic condition with others in their families (especially with respect to participation in support groups or disability communities). However, we did not explicitly ask questions that sought to compare these experiences; so, a conclusion that this aspect of their lived experiences is not salient to genetic and disability identity development is currently unfounded and worthy of further consideration in follow-up studies.

A minority of participants in our study were considered to have atypical cognition and even in these instances, our use of traditional methods of inquiry (survey completion and participation in an interview) were not an impediment to their meaningful participation. Growing the knowledge base for genetic and disability identity development in adolescents with genetic conditions requires the use of transformative, inclusive co-design methods [80, 81]. This will allow for the conduct of research with adolescents whose disabilities present with complex communication profiles who remain excluded from current investigations because of negative perceptions about their capacity to participate [80, 82].

The fluidity we observed in the internalizing processes, and the important role of contextual factors within our conceptual model may have been influenced by the newness of these topics to this group of adolescents and indicate processing of these ideas as the interviews were taking place [58]. This suggests that an examination of these phenomena using a longitudinal study design would be of additional benefit. This would also allow for an investigation of the use of genetic counselling as an intervention to address psychological adaptation and other measures of quality of life in adolescents with genetic conditions.

### Conclusions

Our research provides novel insights about the landscape of genetic and disability identity development and psychological adaptation among adolescents with genetic conditions. Our conceptual model defines three interacting components (i.e., internalizing processes, contextual factors, and external factors) in the complex identity journeys adolescents’ can take to integrate their genetic conditions into their self-construal and social identities. Central to the research is an expansion of the discourse on identity formation through the recognition that adolescents construct both genetic and disability identities, which can be distinct or overlapping. We also demonstrate how measures of psychological adaptation and illness identity (i.e., PAS and IIQ) may be useful clinical tools to assess the state of genetic and disability identity development and determine the need for intervention, especially as it relates to navigating a society that is hampered by ableism. Our findings underscore the need for healthcare professionals including genetic counsellors, educators, and other interested parties to consider ways in which services can be enhanced to address the challenges in navigating these phenomena and facilitate positive identity development among adolescents with genetic conditions.

## Supplementary Information

Additional file 1.

## Data Availability

The datasets generated or analyzed during this study are not publicly available due to privacy/ethical restrictions.

## Abbreviations

UNICEF: United nations children’s fund
GBD: Global burden of diseases
PAS: Psychological adaptation scale
IIQ: Illness identity questionnaire
ANOVA: Analysis of variance
IQR: Interquartile range
NA: Not applicable
FISH: Fluorescence in situ hybridization
WES: Whole exome sequencing
COVID: Corona virus disease
SES: Socioeconomic status

## Footnotes

1 We use the term “apparent” to connote an identity which can be perceived with the senses, and the term “non-apparent” for that which can be concealed from this perception.
