# Correction: Identity development and adaptation in adolescents with genetic conditions: a qualitatively oriented mixed-methods study to develop strategies for optimizing clinical genetics services

**DOI:** 10.1186/s13023-025-04023-5

**Published:** 2025-09-18

**Authors:** Tasha Wainstein, Cyrus Boelman, Connie Ens, William T. Gibson, Kevin Gregory-Evans, Olubayo U. Kolawole, Sheila K. Marshall, Kathryn Selby, Jehannine Austin, Alison M. Elliott, GenCOUNSEL Study

**Affiliations:** 1 Department of Medical Genetics, Faculty of Medicine, The University of British Columbia, Vancouver, BC Canada; 2 BC Children’s Hospital Research Institute, Vancouver, BC Canada; 3 Division of Neurology, Department of Pediatrics, Faculty of Medicine, The University of British Columbia, Vancouver, BC Canada; 4 Pediatric Partnership Heart Program, Pediatric BC Inherited Arrhythmia Program, BC Children’s Heart Centre, Vancouver, BC Canada; 5 Department of Ophthalmology and Visual Sciences, The University of British Columbia, Vancouver, BC Canada; 6 School of Social Work, University of British Columbia, Vancouver, BC Canada; 7 Division of Adolescent Health and Medicine, Department of Pediatrics, Faculty of Medicine, University of British Columbia, Vancouver, BC Canada; 8 Department of Psychiatry, The University of British Columbia, Vancouver, BC Canada; 9 BC Women’s Health Research Institute, Vancouver, BC Canada

**Correction: Orphanet Journal of Rare Diseases 20, 450 (2025).** 10.1186/s13023-025-03968-x

Following publication of the original article [1], the authors reported that the Authorship order regarding the Authors Jehannine Austin and Alison M. Elliott was inadvertently reversed. The corrected order provided in this Correction is accurate.

The original article [1] has been updated.
