# Supplementary material for: Identity development and adaptation in adolescents with genetic conditions: a qualitatively oriented mixed-methods study to develop strategies for optimizing clinical genetics services
Source: Orphanet J Rare Dis. 2025 Aug 21;20:450. doi: 10.1186/s13023-025-03968-x (PMC12372190; doi:10.1186/s13023-025-03968-x)
Supplement: Supplementary file 1 — Additional file 1. [file 13023_2025_3968_MOESM1_ESM.docx]

# Additional File

Full Description of Methodology

### Participants and Recruitment

English-speaking adolescents between 10 and 19 years, living in Canada, who have a confirmed clinical or molecular diagnosis of a genetic condition were eligible to participate in this study. We recruited participants who had previously participated in the Clinical Assessment of the Utility of Sequencing as a Service (CAUSES) study (19), and research conducted by JA’s team. Recruitment sessions were also conducted for healthcare professionals at several specialty clinics within BC Children’s and Women’s Hospitals who then facilitated referrals to the study. Treating physicians who had long-term engagement with the adolescents facilitated referrals of those who they deemed capable of meaningful engagement with the interviews and completion of self-report measures used in this study. We also used posters in clinic waiting areas as well as social media and other web-based platforms (Twitter/X, Instagram, Facebook, and ReachBC) and engaged with support groups and organizations online to recruit patients. The COVID-19 pandemic had a profound impact on recruitment (20), resulting in a prolonged enrolment timeframe of March 2020 to December 2022.

Adolescents and their caregivers were invited to discuss study participation in a “pre-interview” prior to study interviews taking place. The pre-interview facilitated psychological connection with the research team, a space for participants to ask questions about the study and express their preferences regarding logistics; determined the need for accommodations, and allowed participants to specify whether caregivers would be present for the interview (21). When caregivers were expected to be present for interviews, we used this pre-interview meeting as an opportunity to teach them the scaffolding method (a way of helping to facilitate their child’s responses, rather than having them answer on their child’s behalf (21)). Wherever possible, adolescents who were deemed by their caregivers to have capacity, exercised their developing autonomy by consenting for themselves. In the remaining instances, adolescent assent and parental consent were received. Participants were given the opportunity to select their own pseudonyms to protect their anonymity. Additionally, to ensure that participants could not be identified, we opted to report a condition category which reflected the major organ or system impacted by their genetic conditions rather than the name of the condition.

#### Research Team Positionality and Methodological Framework

Our collective perspectives and identities provide an important contribution to our subjective, partial, and situated analysis of adolescents with genetic conditions. We encompass multiple generations (Gen X, millennials), gender identities (cisgender, agender), and a range of backgrounds including both Canadian-born and immigrants (South Africa, UK). The team comprises a diverse group of experienced researchers and clinicians with respect to areas of academic study and clinical practice (genetic counselling, medical genetics, social work, neurology, ophthalmology, cardiology). As such, we position this work within a “clinical epistemology” (22) which focuses on operationalizing knowledge rather than pursuing it for purely theoretical interests. This aligns with our choice of a pragmatic paradigm as the philosophical framework for this cross-sectional study (23,24). We used a convergent (24) and qualitatively-oriented (25) mixed methods design that collected qualitative and quantitative data from our participants simultaneously, analyzed the two data sets separately, and then integrated these findings. Amongst the team, there are those who have a personal or family history of disability/neurodivergence. This manifested in designing a research study that centered participant voices, inclusivity, empowerment, accessibility, and flexibility. Thus, our design prioritized qualitative philosophical assumptions (in this case, constructivism) over their quantitative counterparts.

#### Qualitative Study Design and Data Analysis

Semi-structured interviews took place between participants and TW using the Zoom video-conferencing platform (<https://www.zoom.us>) or in-person according to participant preference. An interpretive description framework was used for the qualitative component (26–28). As interpretive description is a pragmatic approach, it allows for exploration of research questions with a focus on practical implications within a discipline-specific context (28). Interpretive description acknowledges that it is possible to identify broad patterns and shared realities across populations, but also that the variations of those patterns are fractal and endless.

*Data Generation*

We used an interview guide (see Additional File) that we developed based on a scoping review of the literature (8) and the research questions to facilitate discussion. Our approach to interviewing adolescents with genetic conditions was carefully considered to mitigate power imbalances and optimize their feelings of comfort and control and (21,29). Further, in alignment with the core research team’s (TW, JA, AME) professional orientation as genetic counsellors, we incorporated the “remedial pedagogical interviewing” technique to navigate participants’ emotional distress (remedial) and educational needs (pedagogical) when necessary (30,31). Interviews were audio-recorded and transcribed verbatim (by either TW, a professional transcription service, or a research assistant) and were checked for accuracy against the original audio recordings. Transcripts were imported into NVIVO (Release 1.7.1; <https://lumivero.com>) which was used for basic organization and recording of primary-cycle codes and their descriptions.

*Data Analysis Procedure*

We used a phronetic iterative approach to analyze the data (32,33). After immersion in and familiarization with the data through repeated readings of the interviews and reviewing field notes taken after each encounter, TW completed inductive primary-cycle coding for all transcripts. At this point, coding was descriptive in nature and any initial interpretations and reflections were recorded in analytic memos. Using the constant comparative method (34), excerpts for each code were assessed, with modifications or more precise code explanations being iteratively applied as necessary. Initial interpretations of the data were discussed and refined through secondary-cycle coding and collaborative analysis (35) among TW, JA, and AME. As we had not prespecified a theoretical framework at the start, we used theory-informing inductive data analysis (36) to engage with existing literature and theories to provide context to our findings as they arose, and to situate them within broader knowledge bases. Theories identified as being important during the analysis process included theories of human development (37), psychological adaptation (38,39), identity development (40), and disability identity development (41–43). These theories provided a scaffold that reflects a reciprocal relationship between psychological adaptation and identity development and accounts for both contextual factors and the impact of peer relationships and social support networks. Synthesizing (32) was achieved through regular debriefing meetings after each interview and between each stage of the phronetic iterative analysis, the use of field notes and analytic memos, and critical reflections of the ways in which our positionalities and values were influencing our interpretations. The iterative and recursive analysis process and regular meetings allowed for the development and refinement of a conceptual model.

*Quality Practices*

We used information power to evaluate the adequacy of our participant group. The greater the information that is held by the participant group, the lower the number of participants required to address the study aim (44). We used a confirmed clinical or molecular diagnosis of a genetic condition as an important inclusion criterion to ensure specificity which is associated with higher information power. We captured a large amount of variation among our participants with respect to their ages, genetic conditions, cognitive abilities, and whether their conditions were inherited, thereby enriching our study with a diversity of experiences. Information power was further enhanced by having demonstrated resonance of our findings with existing theoretical literature. We evaluated the quality of interview dialogues to be high (skilled interviewer, articulate participants, good rapport between researcher and participants), supportive of higher information power. Finally, we successfully used a cross-case analysis strategy to capture common patterns, variations, and subtlety among the participants, indicating that our participant group provided good information power.

####

#### Quantitative Study Design and Data Analysis

In addition to their interviews, participants completed a brief demographic questionnaire (see Additional File) that included information about age, gender, ethnicity, and genetic condition, as well as two self-report questionnaires. These questionnaires were selected to provide additional insight into adolescents’ overall lived experiences because of their relevance to identity formation and adaptation. All quantitative data were collected and managed using REDCap electronic data capture tools (45,46) hosted at BC Children’s Hospital Research Institute. Demographic information as well as the outcomes of both instruments are reported with respect to their measures of central tendency and distribution.

*Psychological Adaptation Scale*

The first instrument was the Psychological Adaptation Scale (PAS) which assesses emotional and cognitive aspects of coping and adaptation to a condition at a given time (38). Participants responded to 20 statements that reflect their level of agreement with four dimensions that indicate a well-adapted individual: self-esteem; spiritual and existential well-being, coping efficacy; and social integration. Mean (±standard deviation) scores were calculated for each sub-scale (ranging from 1 to 5). An overall score was also calculated by averaging responses across all four dimensions; a score of 3 is considered to reflect adequate adaptation (47), and the higher the score, the higher the level of adaptation to the condition. Cronbach’s alpha (α) was calculated for the total mean PAS as well as each sub-scale. Prior use of the PAS has predominantly evaluated the construct in adults or adult caregivers of children with genetic conditions with results indicating high validity and reliability in populations with neurofibromatosis (39), Rett syndrome (49), bipolar disorder (50), Down syndrome (51), and neuromuscular diseases (48). PAS has also been measured in adolescents with Klinefelter syndrome (39,52).

*Illness Identity Questionnaire*

The second instrument was the Illness Identity Questionnaire (IIQ) which measures the degree to which a condition influences identity formation (48–51). Participants indicated their level of agreement with 25 statements that measured four dimensions of illness identity: rejection (the degree to which a condition is rejected as part of one’s identity); engulfment (the degree to which a condition dominates one’s identity); acceptance (the degree to which a condition is accepted as part of one’s identity); and enrichment (the degree to which a condition positively impacts one’s identity). Mean (±standard deviation) scores were calculated for each sub-scale (ranging from 1 to 5), with higher scores reflecting greater congruence with the dimension. When combined, mean scores of the rejection and engulfment sub-scales provide insight about maladaptive illness integration, while the mean scores of the acceptance and enrichment sub-scales provide insight about adaptive illness integration (52,53). Cronbach’s alpha values were calculated for the four IIQ sub-scales to evaluate internal reliability and consistency in this participant group. The IIQ has shown high levels of validity and internal reliability in populations of adolescents, youth, or emerging adults with type 1 diabetes (54), congenital heart disease (57), neuromuscular disorders (59), celiac disease (60), and refractory epilepsy (58).

####

#### Integration of Qualitative and Quantitative Data

An appropriate way of integrating the qualitative and quantitative analyses emerged by abduction (54,55), a creative inferential process through which we perceive relationships between phenomena and observations. We undertook exploratory inferential statistical analyses to interrogate relationships between our conceptual model and scores from participants’ PAS and IIQ surveys. As our data did not meet assumptions of normality or homogeneity of variance required for parametric tests (i.e., ANOVA), we used the Kruskal-Wallis test, a non-parametric method, to compare medians across the four internalizing processes outlined in the conceptual model. The sub-scales of both the PAS and IIQ, as well as total mean PAS, and the maladaptive and adaptive IIQ scores were ranked across all processes, and the sum of ranks for each process was calculated. The derived test statistic assessed whether ranks differed significantly among the groups. In instances where significant differences were identified, Wilcoxon rank- sum tests were used to determine which process differed from each other. This was achieved by calculating multiple pairwise comparisons of the median differences between any two processes. Statistical significance was defined at alpha level <0.05.

Interview Guide

Introduction: My name is Tasha and I use she/her pronouns. I am a genetic counsellor and a PhD student at UBC. As a genetic counsellor, I talk to families about changes in genes and help give them the information they may need to make decisions. In my work, I have spoken with lots of parents who have children with genetic conditions, but I have realized that I don’t know as much about what it is like for a teenager to have a genetic condition. I think it is important to understand directly from teenagers what their experiences have been. That is why I am doing this research project and why we are having this conversation today. I am really interested to talk to you about what it has been like for you to live with a genetic condition, so thank you so much for agreeing to speak with me. Before we begin, is there anything you’d like to ask me, about either myself, or the research project?

Questions and Prompts:

Let’s start with some basic information about you.

- Can you tell me about your family? Who lives in your house with you?
- How many brothers or sisters do you have? Are they older or younger than you?
- Are you the only person in your family who has [*name of condition*]?

**How do adolescents describe receiving, understanding, and living with a genetic diagnosis?**

- When did you first know that you had [*name of condition*]?
  - *Prompts: Who told you had it; what did they tell you?*
- What do you understand about why you have [*name of condition*]?
- What do you remember about having genetic counselling or genetic testing?
  - *Prompts: What was the experience like; did you attend with your parents; did you speak to the doctor/genetic counsellor alone; what was helpful; what was not helpful?*
- How does your condition impact your family?
  - *Prompts: How does your family talk about [name of condition]; how often does your family talk about [name of condition]; what are your relationships like with your mom/dad/siblings; do you feel like your parents ever treat you differently from your siblings; what sort of differences have you noticed?*
- Who do you talk to when you are worried about [*name of condition*]?
- Beyond your immediate family, who do you talk to about [*name of condition*]?
  - *Prompts: what do you tell them and what do you choose not to tell them?*
- Do your friends know about your condition?
  - *Prompts: If no, how do you think it would change things if people did know; do you think they would think about you differently if they knew; what is it like when people ask you questions about [name of condition]; is it helpful/important when people know the name of your condition?*
- How do you think people see you?
  - *Prompts: How do you describe yourself when you meet someone new; do you ever feel like you are treated differently because of [name of condition]?*

**How do adolescents make sense of their experiences with their genetic conditions?**

- Do you identify as someone with a genetic condition? Is your condition a core part of who you are?
  - *Prompts: Can you tell me more about that; do you think about [name of condition] every day or are there some days that you forget about it; have you ever wished that you didn’t have your condition?*
- What positive and negative emotions have you experienced because of [name of condition]?
  - *Prompts: What do you value about [name of condition]; what do you find difficult about having [name of condition]?*
- How do you see yourself in the future?
  - *Prompts: What excites you about the future; what worries you about the future?*
- What would you compare the experience of having [*name of condition*] to, to convey the meaning it has for you (e.g., graduating from high school; loss of a loved one)?

**What can be learned from adolescents and their ability to adapt to their condition?**

- Do you feel that you belong/fit in with your unaffected family/friends/peers?
  - *Prompts: Do you seek out others who have the same/similar condition; have you connected with a support group; which one; was it helpful; if not, would it be helpful for you to talk with others who have the same or a similar condition; what do your family/friends/school/community do that is helpful?*

**How can clinical services be modified to improve outcomes (e.g. psychological support and adaptation)?**

- How have you found the support from healthcare professionals like your doctors or genetic counsellor?
  - *Prompts: What helps you when you come to the hospital for appointments; what isn’t helpful; what advice would you give to healthcare professionals about the best way to talk to you about [name of condition] or your healthcare needs; In general, have you felt well supported by healthcare professionals?*
- Is there anything else you would like to share about your experience of living with [*name of condition*]?

Demographic Questionnaire

Thank you for agreeing to participate in our research study. The first step is for you to complete three short surveys. Please complete the questions below to the best of your ability.

| How old are you? | years |
| --- | --- |
| What grade are you in at school? |  |
| What pronouns do you use? | she/her  he/him  they/them  other |
| Please specify “other” |  |
| What ethnic group(s) do you most identify with? |  |
| How would you describe your current health?  (i.e., how do you feel today?) | poor  fair  good  very good  excellent |
| How would you describe your general health?  (i.e., how do you feel on MOST days?) | poor  fair  good  very good  excellent |
| What name do you give to your condition? |  |

Psychological Adaptation Scale and Illness Identity Questionnaire Outcomes


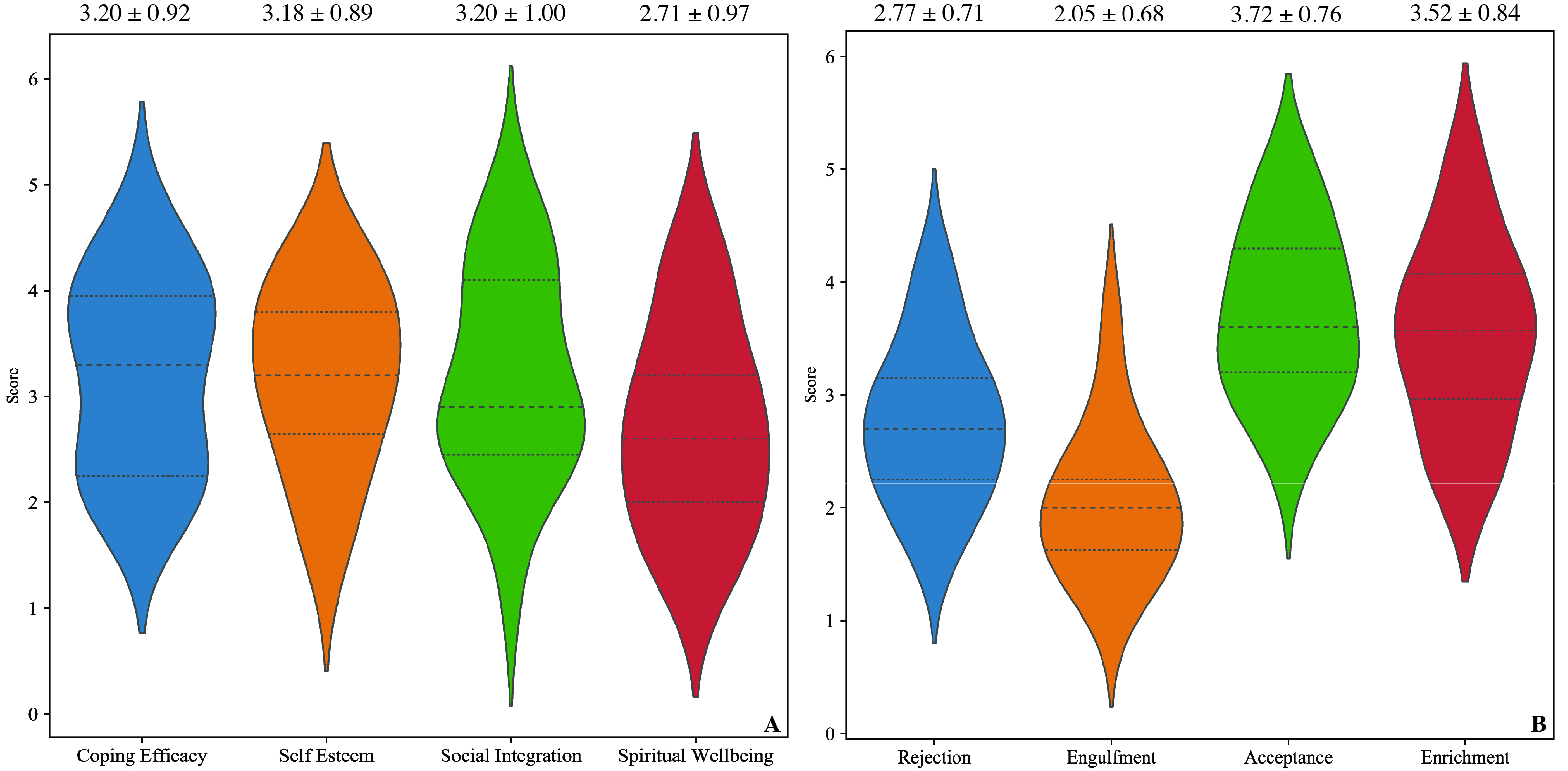


Figure A1 Mean (±SD) and violin plots of the (A) psychological adaptation sub-scales and (B) illness identity questionnaire sub-scales

Statistical Analyses


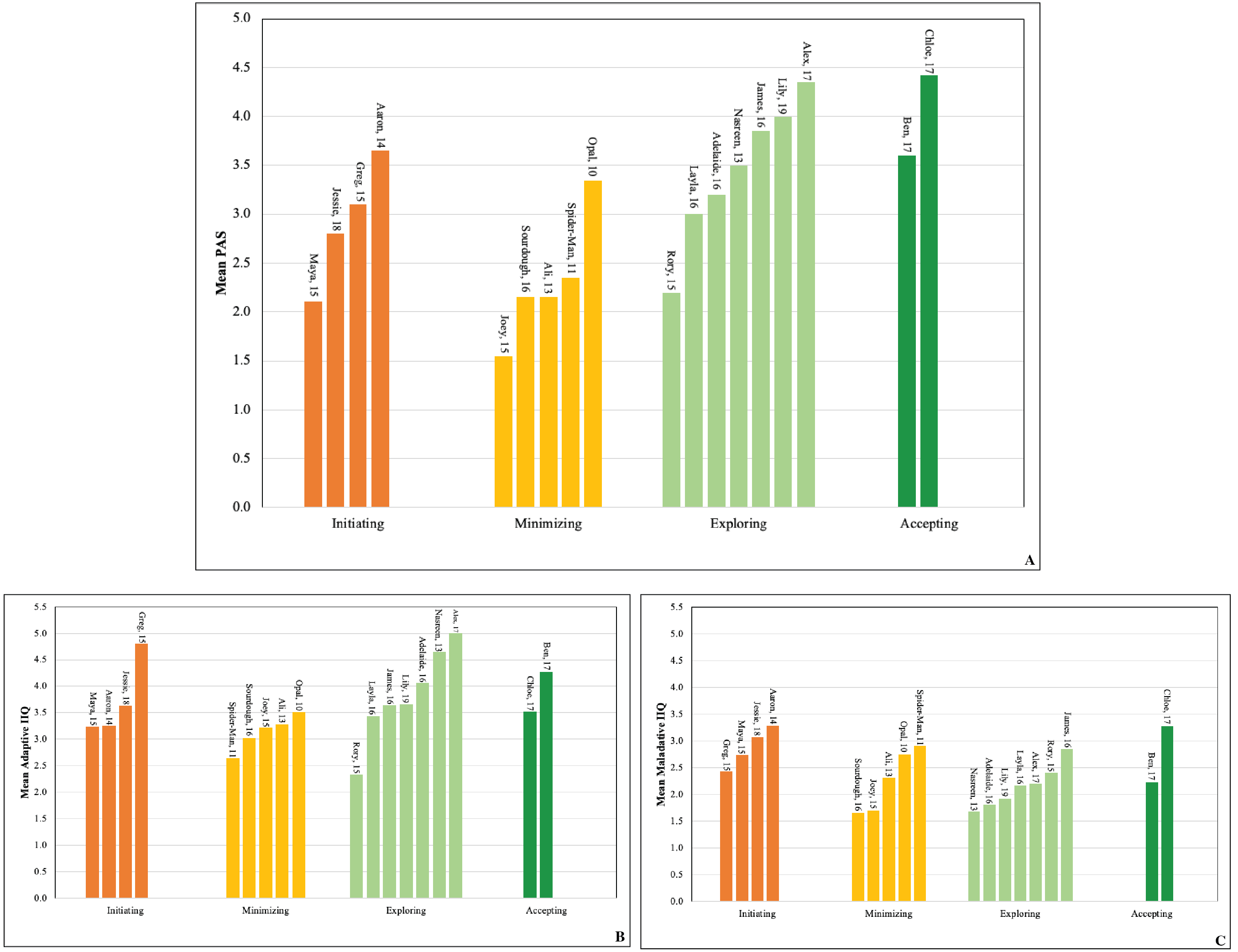


Figure A2 Association of participants’ internalizing processes with (A) total mean PAS, (B) mean adaptative IIQ scores, and (C) mean maladaptive IIQ scores


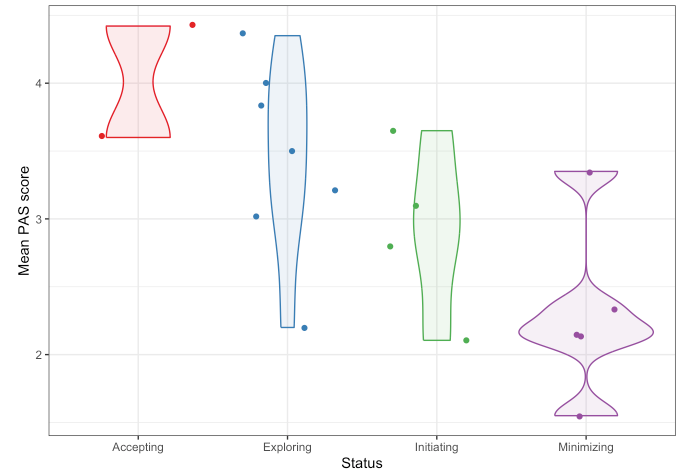


Figure A3 Violin plots of mean PAS scores according to internalizing process.

Table A1 Kruskal Wallis testing for differences in psychological adaptation sub-scales as well as mean total PAS and adolescents’ internalizing processes.

| **Component** | **Statistic** | **P Value** |
| --- | --- | --- |
| Coping efficacy | 8.076198 | 0.0444629* |
| Self esteem | 5.708467 | 0.1266882 |
| Social integration | 8.236841 | 0.0413623* |
| Spiritual | 5.165146 | 0.1600961 |
| Mean total | 7.767414 | 0.0510713 |

Table A2 Pairwise Wilcox tests for PAS mean total score by internalizing process pairs.

| **Group 1** | **Group 2** | **P Value** |
| --- | --- | --- |
| Exploring | Accepting | 0.9212121 |
| Initiating | Accepting | 0.9212121 |
| Initiating | Exploring | 0.9212121 |
| Minimizing | Accepting | 0.3932145 |
| Minimizing | Exploring | 0.2066333 |
| Minimizing | Initiating | 0.9212121 |

Table A3 Pairwise Wilcox tests for PAS coping sub-scale by internalizing process pairs.

| **Group 1** | **Group 2** | **P Value** |
| --- | --- | --- |
| Exploring | Accepting | 0.7187963 |
| Initiating | Accepting | 0.4008380 |
| Initiating | Exploring | 0.7187963 |
| Minimizing | Accepting | 0.3525401 |
| Minimizing | Exploring | 0.1322454 |
| Minimizing | Initiating | 1.0000000 |

Table A4 Pairwise Wilcox tests for PAS social integration sub-scale by internalizing process pairs.

| **Group 1** | **Group 2** | **P Value** |
| --- | --- | --- |
| Exploring | Accepting | 0.5946263 |
| Initiating | Accepting | 0.3808188 |
| Initiating | Exploring | 0.4577806 |
| Minimizing | Accepting | 0.3796348 |
| Minimizing | Exploring | 0.3054789 |
| Minimizing | Initiating | 0.5946263 |


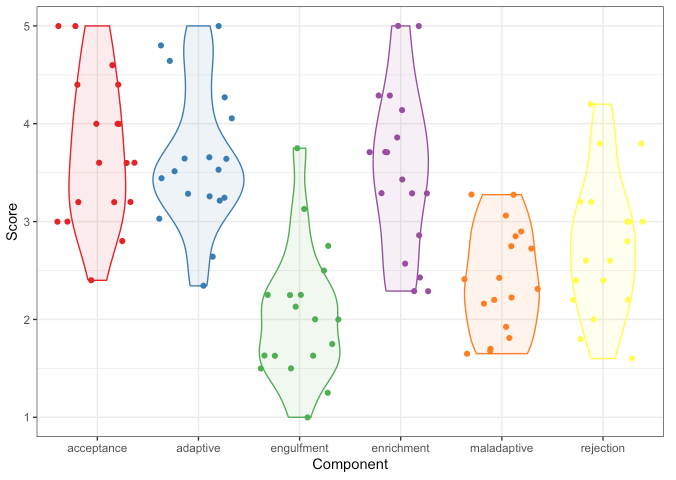


Figure A4 Violin plots of IIQ sub-scale, adaptive, and maladaptive scores according to internalizing process.

Table A5 Kruskal Wallis testing for differences in IIQ sub-scales as well as adaptive and maladaptive scores and adolescents’ internalizing processes.

| **Component** | **Statistic** | **P Value** |
| --- | --- | --- |
| Rejection | 2.546016 | 0.4670324 |
| Acceptance | 0.407944 | 0.9385966 |
| Engulfment | 9.697825 | 0.0213173* |
| Enrichment | 6.543897 | 0.0879473 |
| Maladaptive score | 5.761397 | 0.1238138 |
| Adaptive score | 5.337275 | 0.1487019 |

Table A6 Pairwise Wilcox tests for IIQ engulfment sub-scale by internalizing pairs.

| **Group 1** | **Group 2** | **P Value** |
| --- | --- | --- |
| Exploring | Accepting | 0.2800330 |
| Initiating | Accepting | 1.0000000 |
| Initiating | Exploring | 0.0622746 |
| Minimizing | Accepting | 0.5714286 |
| Minimizing | Exploring | 1.0000000 |
| Minimizing | Initiating | 0.2800330 |
